# Does Neutrophil Phenotype Predict the Survival of Trauma Patients?

**DOI:** 10.3389/fimmu.2019.02122

**Published:** 2019-09-06

**Authors:** Esmaeil Mortaz, Seyed Sajjad Zadian, Mehri Shahir, Gert Folkerts, Johan Garssen, Sharon Mumby, Ian M. Adcock

**Affiliations:** ^1^Department of Immunology, Faculty of Medicine, Shahid Beheshti University of Medical Sciences, Tehran, Iran; ^2^Clinical Tuberculosis and Epidemiology Research Center, National Research Institute of Tuberculosis and Lung Diseases, Shahid Beheshti University of Medical Sciences, Tehran, Iran; ^3^Division of Pharmacology, Faculty of Science, Utrecht Institute for Pharmaceutical Sciences, Utrecht University, Utrecht, Netherlands; ^4^Nutricia Research Centre for Specialized Nutrition, Utrecht, Netherlands; ^5^National Heart and Lung Institute, Imperial College London, London, United Kingdom; ^6^Priority Research Centre for Healthy Lungs, Hunter Medical Research Institute, The University of Newcastle, Newcastle, NSW, Australia

**Keywords:** trauma, neutrophil subtype, injury, survival, neutrophils

## Abstract

According to the World Health Organization (WHO), trauma is responsible for 10% of deaths and 16% of disabilities worldwide. This is considerably higher than those for malaria, tuberculosis, and HIV/AIDS combined. While the human suffering and death caused by injury is well-recognized, injury has a significant medical care cost. Better prediction of the state of trauma patients in the days immediately after trauma may reduce costs. Traumatic injuries to multiple organs can cause dysfunction in all systems of the body especially the immune system placing patients at high risk of infections and inflammatory complications which are often fatal. Neutrophils are the most abundant leukocyte in the human circulation and are crucial for the prevention of microbial disease. Significant changes in neutrophil functions such as enhanced chemotaxis, Neutrophil extracellular trap (NET)-induced cell death (NETosis), and phagocytosis occur early after injury followed by prolonged functional defects such as phagocytosis, killing mechanisms, and receptor expression. Analysis of these changes may improve the prediction of the patient's condition over time. We provide a comprehensive and up-to-date review of the literature investigating the effect of trauma on neutrophil phenotype with an underlying goal of using this knowledge to examine the predictive potential of neutrophil alterations on secondary complications in patients with traumatic injuries. We conclude that alterations in neutrophil surface markers and functions may be potential biomarkers that predict the outcome of trauma patients.

## Introduction

Neutrophils are the most abundant leukocyte in humans (60–70% of circulating leukocytes) and have a major role in the innate immune response against invading pathogens and are important mediators of inflammation-induced injury (1). In healthy adults, circulating neutrophils are considered as dormant cells but they are activated when they encounter damage-associated molecular patterns (DAMPs) or pathogen-associated molecular patterns (PAMPs) and thereby maintain homeostasis within the immune system (2).

**Figure 1:**
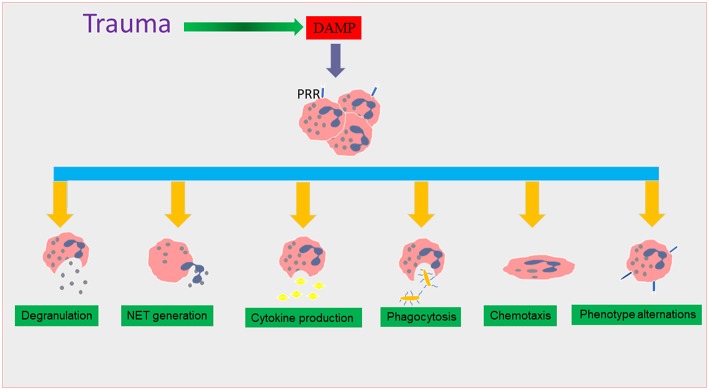
The effect of trauma on neutrophil functions. During traumatic injury, damage associated molecular patterns (DAMPs) are released into the systemic circulation as a result of tissue damage. DAMPs interact with surface pattern recognition receptors (PRR), causing neutrophil recruitment which triggers many functional responses that are thought to lead to the induction of the systemic inflammatory response (SIRS) phase in trauma. These neutrophil phenotype changes include alterations in degranulation, neutrophil extracellular trap (NET) formation, cytokine production, and chemotaxis and phagocytosis.

Neutrophils are equipped with a range of anti-microbial mechanisms including phagocytosis, degranulation, and release of reactive oxygen species (ROS), neutrophil extracellular traps (NETs), and cytokine production that deliver lethal hits against microorganisms (3, 4). Neutrophils rapidly adapt to changes in microenvironmental signals and show different functional phenotypes (neutrophil heterogeneity) during inflammation (5, 6). Trauma affects the phenotype and function of neutrophils (7) although similar changes in neutrophil phenotype have been observed with acute coronary syndromes and in patients with the acute phase of autoimmune disease [Figure 1; (8, 9)]. The World Health Organization (WHO) estimated that trauma causes 5.8 million deaths annually (10, 11) although mortality rates related to trauma have significantly reduced in recent years due to improvements in treatment particularly in those treating coagulopathy and blood loss. However, secondary complications, such as sepsis, multiple organ failure (MOF), and nosocomial infections can impact upon the condition of trauma patients and result in death (12). The induction of altered immune system responses particularly changes in neutrophil phenotypes is increasingly recognized as an important factor in the response to trauma (7).

Marked alterations in a range of neutrophil functions and in phenotypic markers occur following trauma, which causes a massive release of neutrophils, banded cells and sometimes even immature cells (i.e., metamyelocytes) from the bone marrow into the circulation (13, 14). Experimental studies strongly suggest a direct relationship is present between trauma severity and subsequent tissue damage and neutrophils dysfunction (15). In addition, impaired neutrophil chemotaxis, due in part to release of immature neutrophils into the circulation, was seen in children following blunt force trauma (16).

In this review we provide a comprehensive overview of the most recent studies examining the role of neutrophils in severe trauma with the goal of identifying a link between trauma severity and neutrophil phenotype and function. This knowledge of the correlation between neutrophil phenotype and the prediction of patient survival may provide better early biomarkers for the clinical outcome and treatment of trauma patients.

## The Mechanisms of Immune System in Trauma

Trauma activates innate immune responses to produce pro- and anti-inflammatory cytokines primarily by cells of the innate immune response such as neutrophils. The systemic inflammatory response syndrome (SIRS) and the Compensatory Anti-inflammatory Response syndrome (CARS) may be induced with severe trauma without being accompanied by sepsis and multiple organ failure (17). CARS is considered an imprecise term that does not truly reflect the key role that the neutrophil plays in immune tolerance during trauma. The multifunctional aspects of neutrophil biology in this process is critical to induction of immune tolerance by acting as danger- or damage-sensing cells in multiple organs which reflects their Janus-like effects in trauma (17).

While SIRS is a pro-inflammatory syndrome that is associated with killing infectious organisms through activation of the immune system, immune tolerance represents is a complex pattern of immunologic responses characterized by deactivation of the systemic immune system. As such, immune tolerance is not simply the reversal of SIRS, but it can exist separately from SIRS. Immune tolerance can be dangerous when its effects are unchecked, leaving the host vulnerable to a secondary exposure to pathogens because of increased immune effector cell apoptosis leading to auto-immunity (18, 19). Studies have also shown that immune tolerance reduces the severity of the SIRS pro-inflammatory response, but after trauma, tolerance can lead to increased immunosuppression (17). The uptake of apoptotic cells by dendritic cells and macrophages promotes tolerance by suppressing the release of pro-inflammatory cytokines, and increasing the release of anti-inflammatory cytokines, such as IL-10 and transforming growth factor-β (TGF-β) (20, 21).

**Figure 2:**
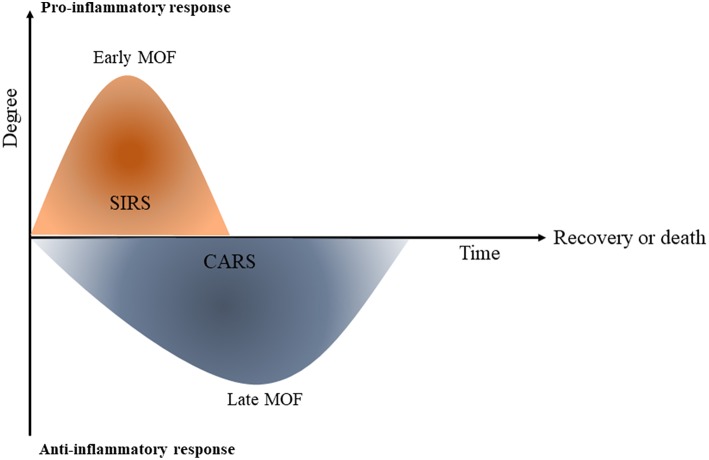
Systemic inflammatory response (SIRS) and compensatory immune tolerance in trauma (CARS). Schematic diagram shows that once trauma has occurred, a primary systemic pro-inflammatory response (SIRS) is initiated which can contribute to early multi-orgn failure (MOF). The compensatory immune tolerance or anti-inflammatory response syndrome (CARS) can begin while the pro-inflammatory SIRS is still present. At the later phase CARS can lead to immune paralysis and following that, late multi organ failure [This figure is adapted from Hietbrink et al. (23)].

One of the important factors that increases morbidity in post-traumatic cases is an imbalance between the systemic inflammatory response SIRS and tolerance (22). In the first hours of trauma, the severity of the SIRS is associated with early MOF and infections however, in the following days, immune tolerance plays an important role in the increased incidence of nosocomial infections and late organ failure and late sepsis [(23); Figure 2].

## Neutrophil Apoptosis in Trauma

A number of studies propose that dysregulated apoptotic immune cell death may have an important role in the severity of multiple organ dysfunction and sepsis following a variety of traumatic events (24, 25). Understanding the role of altered apoptotic cell death in contributing to immune and organ dysfunction as seen in sepsis and shock is essential. Apoptosis is increased following various traumas such as ischemia, burn, sepsis, and traumatic brain injury (26–28). The incidence of programmed cell death following traumatic brain and spinal injury has been studied and re-enforced the importance of apoptosis in the pathogenesis of post-traumatic outcomes although no data yet relates this to neutrophil function (26–28).

Hypoxia can induce apoptosis in various organs, but studies have shown no increase in apoptotic liver cells in trauma animals, despite the presence of systemic hypoxia. Therefore, it is likely that local mechanisms are responsible for the induction of apoptotic cell death (29, 30). Oxidative stress, ischemia, and some mediators such as steroids, tumor necrosis factor (TNF), nitric oxide, complement C5a, and Fas ligand (FasL) have been identified as important factors that induce apoptotic cell death (31–33). Caspase 8 is activated mainly by extracellular signaling through the Fas/FasL system (CD95/CD95L) or via TNF-α receptors suggesting that the reported enhanced expression of caspase 8 indicates an extracellular stimulation of apoptosis (34–36). Immune cells that undergo modified apoptotic changes include neutrophils, macrophages, dendritic cells, and lymphocytes although epithelial and endothelial cell apoptotic changes have also been reported (37). Although neutrophils are conventionally thought of as short-lived cells which die following apoptosis, they may persist for several days (38, 39), and whilst most studies focus on programmed cell death following different traumatic insults they do not determine the mechanisms of neutrophil accumulation at the region of interest (38, 39).

## Neutrophil Apoptosis in Sepsis and Shock

The initial role of neutrophils is innate defense against infection by eliminating pathogens. They kill pathogens using reactive oxygen species (ROS) and lytic enzymes, so they can potentially contribute to bystander organ injury. Therefore, neutrophil apoptosis reduces neutrophil-mediated tissue damage (40). Activation of neutrophils, which induced by trauma, is directly linked to the immune response. Trauma is usually associated with over-activation of innate immune responses followed by a further immune-suppression, which leads to elevated susceptibility to infection, sepsis, and multiple organ dysfunction syndrome (MODS) and act as one of the leading causes of human death (23, 41–43).

Inflammatory mediators may prolong the circulation half-life of neutrophils from 6 h up to several days based on the decreased level of pro-apoptotic proteins, including apoptosis regulator Bax and upregulation of anti-apoptotic proteins, such as myeloid cell leukemia 1 (Mcl-1) (44–46). Delayed apoptosis of neutrophils induces tissue damage by the release of ROS and neutrophil elastase (NE) (47, 48). Inhibition of neutrophil apoptosis has been reported in SIRS (49), sepsis (46, 50), and burn injuries (51).

In shock or injury, exposure to pro-inflammatory factors are thought to prime circulating neutrophils and induce tissue injury (52, 53). Studies have shown that following hemorrhage or endo-toxemia, a remarkable decrease in lung neutrophil apoptosis has been seen for up to 24 h after the insult (54). Jimenez et al. showed a significant decrease in apoptosis in SIRS patients and that this resulted in amplified neutrophil-mediated killing which, in a non-inflammatory environment, may result in SIRS and subsequent organ failure. Therefore, suppression of neutrophil apoptosis increases the potential for tissue injury (55). It is unclear whether the death receptor or mitochondrial-mediated, apoptotic pathway predominates in controlling neutrophil apoptosis in animals exposed to septic shock (56). Studies of neutrophil apoptosis in sepsis cases demonstrated that delayed apoptosis seems to occur as a result of the activation of anti-apoptotic factors and NF-κB and then suppression of caspases 9 and 3 (57–62).

The development of sepsis after major trauma is associated with changes in the expression of apoptosis-related factors (63). After trauma, in the early phase, neutrophil apoptosis is mainly regulated by anti-apoptotic B-cell lymphoma 2 (Bcl-2) members that inhibit the intrinsic mitochondrial-dependent pathway (63). However, neutrophil apoptosis is not always associated with the expression of the anti-apoptotic factor Mcl-1. Neutrophils in patients with sepsis 10-days after the initial trauma displayed reduced neutrophil apoptosis despite decreased levels of Mcl-1 and the Bcl-2-associated A1 protein. The association between reduced neutrophil apoptosis and the severity of illness supports the importance of neutrophils activity in the pathophysiology of sepsis (63).

The soluble form of Fas (sFas) is derived by alternative splicing from the membrane form or by proteolytic cleavage of membrane-bound receptors. Serum sFas has been shown to inhibit neutrophils apoptosis *in vitro* (64) and plays an important role in the inhibition of neutrophil extrinsic apoptosis associated with increased levels of polymorphonuclear leukocyte elastase (PMNE), a marker for systemic inflammation. The results show a high relationship between sFas and patients' Sequential Organ Failure Assessment (SODA) and Multiple Organ Dysfunction (MOD) stage in sepsis and provide evidence for the clinical significance of the risk for the development of sepsis and MOF. Trauma patients with and without sepsis development demonstrated a significant reduction in the apoptosis of circulating neutrophils at least until 10-days after trauma. So, sFas may be a feasible target for new therapeutic strategies to limit neutrophil life span and hyperactivity (65).

## NETs and Organ Injury

NETs may have an important role in the regulation of inflammatory responses to injury. Accumulation of activated neutrophils occurs in the damaged tissue following injury and these may form NETs (66). Recent studies have shown the potential role of NETs in the pathogenesis of an extensive range of non-infectious inflammations including post-injury sterile inflammation (67). Margraf and co-workers in 2008 showed that NETs levels in plasma may predict sepsis and MOF on the intensive care unit in patients after multiple trauma (68, 69).

The severity of tissue damage in cases of transfusion-related acute lung injury (TRALI) is associated with the degree of NETs formation with NETs detectable in the plasma and lung of TRALI patients (70). Mitochondrial DNA can trigger NETosis via activation of Toll Like Receptor (TLR)9 after severe trauma, independent of the NADPH oxidase system (71) and mitochondrial (mt)DNA is found in NETs formed after trauma (66). The detailed molecular mechanism of mtDNA-NETs release is unknown (67).

As NETs are rapidly degraded by DNase in the circulation, it is possible that NETs are actively produced throughout the 5-days after trauma and surgery (72).

However, surgery alone can stimulate NETs formation independent of prior trauma as evidenced by NETs formation after elective total hip replacement. This suggests that sepsis may not have been an initiating factor for the NETs formation. NETs formation in these patients can be viewed as part of the sterile inflammatory response of the innate immune system (72).

The importance of neutrophil-neutrophil cross-talk and connection with other cells related to NETs formation has been shown. Platelets are the most well-defined players in NETosis. Many platelet-derived soluble factors and ligand/receptor pairs maintain neutrophil activation (73). Among these soluble factors, alarmins such as platelet-derived high-mobility group protein box 1 (HMGB1) and chemokines including platelet factor 4 (PF4)/CXCL4) produced by platelets activate neutrophil NETs formation *in vitro* and in animal models (74).

In human neutrophils, P-selectin may drive sterile NETs formation (74). Other platelet-localized cell adhesion molecules such as β2 integrin (CD18) may also play a critical role in this process (74, 75). Indeed, platelet biology impacts upon many aspects of inflammation which makes the identification of their direct or indirect contribution to NETosis not readily predictable (67).

Improved methodologies are needed for the better understanding of detailed mechanisms of NETs. The current techniques combine fluorescent microscopy or fluorescent intensity measurements and generally use DNA-intercalating dyes, while taking the risk of visualizing necrotic cells with dye permeable cell membrane. Antibody-based techniques are required to detect activated, non-necrotic cells with intact cell membrane, such as flow cytometry-cell-sorting, supported by microscopic imaging. Additionally, a consensus on the structural and behavioral definition of NETs formation is essential for future NETs research, due to their fragility, their highly dynamic nature and their morphological heterogeneity (67).

## Trauma and Modulation of Neutrophil Phenotype

Within hours and days after trauma, the expression of neutrophil markers become noticeably distinct compared to those from healthy individuals. The various markers show distinct dynamics over time. In this regard, the severity of changes in function and phenotype of neutrophils in trauma depends upon the severity of injury as measured by indices such as the injury severity score (ISS) and the new injury severity score (NISS) (76, 77).

Some studies report a significantly higher percentage of neutrophils positive for CD11a in trauma patients than in controls at 3 and 96 h after injury (78). However, another study showed that there is a significant decrease in CD11a expression 24 h after trauma (79). Integrins (e.g., Mac-1 also known as alpha M beta 2 integrin which is composed of CD11b and CD18) are involved in leukocyte adhesion to the endothelium. Functional integrins are only expressed upon neutrophils activation (80) and neutrophil Mac-1 expression has been proposed as a marker of injury severity in several studies (81–83). Circulating neutrophils show upregulation of CD11b, after injury with a second peak of CD11b expression at day 5 (79). Contrarily, Scannell and colleagues have reported attenuation in expression of ICAM-1, CD11b, and CD18 on circulating leukocytes 2 h after injury (84). The level of metabolic acidosis after trauma correlates directly with CD11b expression on circulating neutrophils which may provide a mechanism whereby post-traumatic shock results in neutrophil-mediated organ failure (82).

Increased Mac-1 expression is found on neutrophils from patients who were admitted with an ISS >16 as compared to trauma patients with an ISS <16 which could be a useful marker for prediction of survival of trauma patients but need more investigation (78). In addition, neutrophils sampled at various time points pre- and post-operative days had increased expression of CD11b when treated with Platelet-activating factor (PAF) and/or fMLP (N-formyl-methionyl-leucyl-phenylalanine (fMLP) although the expression of CD11b in unstimulated conditions did not change with surgery, suggesting minimal activation *in vivo* and a failure of PAF to act as an agonist on human neutrophils (85).

CD18 expression follows the same expression pattern following trauma as CD11b although this did not reach significance (78). However, similar to the *in vitro* findings, the numbers of CD11a surface receptors do not increase synchronously with CD11b/CD18 receptors although their affinity may increase (78).

The expression level of CD62L (L-selectin), a receptor that mediates the initial step of the adhesion cascade, the capture and rolling of leukocytes on endothelial cells, was decreased up to 24 h after injury (85, 86). No correlation was demonstrated between immune cell CD62L expression and trauma severity scores although a meta-analysis by Stengel and colleagues indicated that soluble L-selectin levels were correlated with ISS (87). Some studies have reported an association between decreased L-selectin expression on leukocytes and the occurrence of SIRS or early MOF. These studies also show a correlation between the degree of neutrophils activation and the severity of complications occurring during the pro-inflammatory phase (88, 89). These molecules can be found as soluble factors in serum (sL-selectin). The level of sL-selectin in the blood is correlated with the activation level of the neutrophil population (23).

Compared to control individuals, traumatic patients were characterized by a statistically significant decreased responsiveness of active FcγRII (a marker of neutrophil priming) on neutrophils toward the fMLP (90, 91). High levels of active- FcγRII expression was indicative of increased responsiveness to bacterial products (92). The decreased responsiveness of active FcγRII toward the fMLP on the circulating neutrophils after trauma may impact on downstream inflammatory events. Indeed, the degree of this reduced responsiveness correlated with trauma severity as measured by ISS (91). Furthermore, the degree of decrease in fMLP-induced active FcγRII on neutrophils is related to severity of the clinical response and to SIRS (91).

In contrast, the expression of FcγRIII (CD16) on neutrophils was suppressed during severe trauma. Furthermore, soluble CD16 increased significantly at day 1 in multi-trauma patients who later developed infection (93). Similar results were also seen during the first 24 h after chest trauma (92). FcγRIII is normally expressed on banded neutrophils at lower levels compared with mature neutrophils. Therefore, this decline in FcγRIII may reflect an influx of young neutrophils (90).

CD43 (Leukosialin) is expressed on early hematopoietic progenitors and is one of the most abundant transmembrane sialoglycoproteins on neutrophils (86). CD43 prevents interactions of surface molecules and acts as a negative regulator of cell function (94). CD43 membrane expression was decreased by up to 80% upon exposure to phorbol-12-myristate-13-acetate (PMA) or fMLP in the presence of cytochalasin B (95). PMA activation significantly reduced neutrophils CD43 expression. Downregulation of CD43 has been seen in hemodialysis, neutrophil activation, and during neutrophil migration whereas, fMLP normally causes CD43 shedding from neutrophils (96).

## Impact of Trauma on Neutrophil Migration

The key regulatory step in neutrophils tissue migration occurs at the level of bone marrow release and association with the endothelial layer (23). There is a large storage pool of mature neutrophils in the bone marrow which is rapidly mobilized during inflammation causing a dramatic rise in circulating neutrophil numbers (97). CXCR4 and its ligand, CXCL12 (or stromal cell derived factor-1), trigger neutrophils release from the bone marrow under normal conditions (98). In addition, CXCL12 acts as a chemokine by attracting neutrophils to the site of inflammation (99) and not surprisingly, therefore, activated CXCR4 has been described as having multiple biological functions including chemotaxis, differentiation and survival (100).

During trauma, the release of DAMPs enhances neutrophils migration through the 600 μm sinusoids and they then elongate and squeeze themselves through the tissue to reach the site of injury (101, 102). High mobility group box-1 (HMGB-1), a recently identified inflammatory cytokine, is implicated in the pathogenesis of several inflammatory diseases where it acts as an important DAMP (103). The interaction of HMGB1 with specific receptors on numerous cell types results in increased production and release of proinflammatory cytokines and chemokines (104). In some studies, HMGB-1 expression correlates with ISS (105) but this is not replicated elsewhere (106). In light of these contradictory data, it is important to examine the potential of HMGB-1 as a biomarker of trauma in much larger homogenous cohorts and after therapeutic intervention.

Generally, the leukocyte recruitment cascade contains the following steps: (a) selectin-mediated rolling, (b) chemokine-triggered activation, and (c) integrin-dependent arrest (107). Rolling is mediated by L-selectin (CD62L), P-selectin (CD62P), and E-selectin (CD62E), which interact with glycosylated ligands such as P-selectin glycoprotein ligand 1 (PSGL1), ESL1 (E-selectin ligand 1), and CD44 (108). The interaction of selectins with their ligands enables leukocytes to adhere to the inflamed endothelium (109). Consequently, down regulation of L-selectin expression on leukocytes and induction of SIRS or MOF, may suggest cross talk between the development of complications occurring during SIRS and the degree of neutrophils activation (91, 92). Importantly, the serum levels of sL-selectin are higher 6 h after trauma during which time neutrophils migrate to the tissue (78).

**Figure 3:**
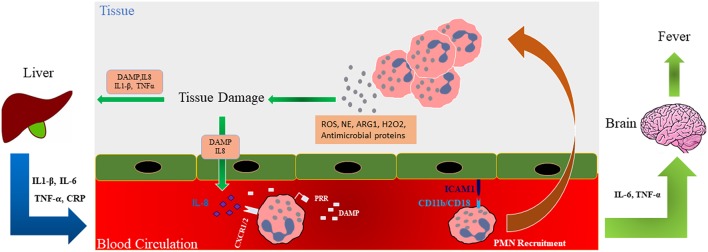
Role of neutrophils in tissue damage. Schematic illustration shows that during trauma, damage associated molecular patterns (DAMPs) are generated and these along with released mediators (ROS; NE; ARG1; hydrogen peroxide, H_2_O_2_, and antimicrobial proteins) recruit neutrophils to the site of injury where they release factors such as additional ROS and NE involved in the development of organ failure in a feedforward manner. Interleukin (IL)-6 and TNF-α can affect the brain causing fever in traumatic patients. Trauma-generated DAMPs also affect the liver and cause the release of inflammatory cytokines into the circulation which, in turn, further modulate neutrophil numbers and activation. DAMP, Damage-associated molecular pattern; ROS, Reactive oxygen species; PRR, pattern recognition receptor; ICAM-1, Intercellular Adhesion Molecule 1; ARG1, Arginase 1; NE, Neutrophil elastase; TNF-α, Tumor necrosis factor alpha.

High concentrations of the neutrophil chemotactic factor IL-8 have been reported in trauma patients (110, 111). IL-8 activates and recruits neutrophils to the site of inflammation by interacting with its receptors CXC receptor 1 (CXCR1) and CXCR2 on the neutrophils cell membrane [Figure 3; (112)]. CXCR1 responses are lower in trauma patients than in control subjects but the activity of CXCR2 is higher and may be implicated in the later clinical complications seen with neutrophil activation. Indeed, CXCR2 activity correlates with neutrophils hyperactivity and with outcomes such as acute respiratory distress syndrome (ARDS) whereas reduced CXCR2 function seen in inflammatory environments may impair neutrophil functions (46, 113, 114). Activation of circulating endogenous factor VII-activating protease (FSAP) in multiple trauma patients led to increased complement (C)5a anaphylatoxin generation and modulation of the posttraumatic SIRS *in vivo* (115). C5a is a potent chemoattractant involved in the activation and recruitment of neutrophils at the site of trauma (116). Robust C5a generation during trauma may cause defects in neutrophil defense systems and C5a might be considered as a potential target for therapeutic intervention to prevent immune dysfunctions that occur in the days following trauma (117).

Integrins are important components of the transmigration process and are only expressed on activated neutrophils (80). Thus, the expression of Mac-1 is increased on neutrophils from multi trauma patients as compared to mild trauma patients suggesting neutrophil activation (78). In contrast, during late organ failure the expression of Mac-1 is down-regulated (118) and is associated with the development of MOF (119).

## Impact of Trauma on Neutrophil Activity

The formation of free radicals and ROS is an important component of activated neutrophils following trauma and is involved in phagocytosis.

### Oxidative Stress

Neutrophils are the major cellular producers of ROS and alterations in the levels of ROS production reflect neutrophils activation status. The production and role of the oxidative burst in neutrophils during brain trauma has been extensively reviewed (120–122). Increased oxidative burst correlates with the incidence of SIRS and MOF (123). On the other hand, ROS have been identified as a necessary component of the NLRP3 inflammasome activator in various diseases including hepatic ischemia/reperfusion injury (124). The NLRP3 inflammasome is essential for the onset of acute sterile inflammation such as that seen in trauma injury (125).

Increased ROS production by neutrophils may lead to an uncontrolled inflammatory response which results in tissue damage. The highest levels of ROS production were found between 3 and 24 h after trauma after *in vitro* stimulation of the cells (126). Furthermore, the expression of inducible nitric oxide (NO) synthase (iNOS or NOS2) and of NADPH oxidase (gp91phox) in leukocytes is reduced following traumatic brain injury (TBI), whereas, after 24 h the expression of iNOS, cyclooxygenase (COX)-2 and of gp91phox is significantly increased in monocytes and neutrophils (127). This leads to increased prostaglandin (PG) induction either directly or indirectly. Spinal cord injury (SCI) causes a systemic inflammatory response resulting in increased oxidative burst within neutrophils and monocytes from 12 to 48 h and 1 week post-injury and the maximal increase was at 24 h in neutrophils. In addition, the expression of gp91phox, COX-2 and iNOS were significantly increased 24 h after trauma in neutrophils and monocytes of these patients (128). Furthermore, the newly formed ROS in injured tissue leads to the migration and subsequent activation of neutrophils resulting in the accumulation of activated neutrophils in the spine (129). Table 1 indicates the markers that reflect changes in neutrophil functions/phenotype of neutrophil biology that occur during trauma.

**Table 1:** Functions and markers that reflect changes in neutrophil functions/phenotype in trauma.

**Function**	**Description**	**Increased level**	**Decreased level**
Priming	Result of exposure to priming agents such as GM-CSF and TNF-α	Result to enhanced functions of neutrophils (chemotaxis, adhesion, rolling, and oxidative burst) (130)	
Rolling	Mediated by selectins	Level of sL-selectin indicates number of neutrophils which migrate to the tissue (78)	Decreased L-selectin on neutrophils show incidence of SIRS or early MOF (88)
Adhesion	Integrins are involved in the adhesion of leukocytes to the endothelium	High expression of Mac-1 related with the development of SIRS and organ failure (119)	Neutrophil Mac-1expression was decreased during late organ failure from patients who died from the consequences of sepsis as compared to patients who survived (118)
Oxidative burst	Necessary for pathogen killing by neutrophils	Increased oxidative burst correlates with the incidence of SIRS and MOF (123)	Low oxidative burst is related with sepsis.
Apoptosis	Delayed neutrophil apoptosis is seen after trauma		Delayed apoptosis results in accumulation of neutrophils and promote tissue damage (47, 48)
Other	HMGB-1	Increased levels of HMGB-1 disrupts endothelial barrier function and recruitment of neutrophils (131)	
	CD64	CD64 expression level on day 1 is a fair predictor of outcome in critically ill patients with severe trauma and/or severe sepsis (132)	
	cf-DNA/NETs	The levels of cf-DNA/NETs in serum is increased in trauma patients who subsequently develop sepsis (133)	
	CXCR2	CXCR2 activity correlates with neutrophil hyperactivity and with outcomes in ARDS (113)	Reduced CXCR2 function in inflammatory environments impair neutrophil function (113)
	C5a	Robust C5a generation during trauma may cause defects in neutrophil defense systems (117)	
	Responsiveness to fMLP		Decreased ability of fMLP to induce active FcγRII on neutrophils in patients with secondary complications of trauma (91)

## Phagocytosis and Killing

A fundamental step in the host defense response against infection is phagocytosis (134). Given that perturbation in immune responses followed by multi trauma may lead to sepsis, investigation of neutrophils phagocytosis seems necessary. Changes in neutrophils phagocytic activity following trauma vary depending on the microbial species which explain the conflicting results observed with phagocytic kinetics (135–137).

Some reports have demonstrated decreased neutrophil phagocytosis following trauma and injury, for example, the lowest rates of neutrophil phagocytosis were observed at 48 and 72 h after TBI (127). In contrast, the neutrophil phagocytosis index (FI) was higher 1–3 days after severe tissue injury (138). Generally, after trauma, ingestion of E *coli* was enhanced, whereas phagocytosis of K *pneumoniae* was depressed. Ingestion of S *aureus*, however, was unaffected (134). This effect may also involve platelets which also express TLR2 ns TLR4 enabling the generation of NETs and the killing of gram positive and negative bacteria (139).

## Neutrophil Life Span During Trauma

The lifespan of circulating neutrophils is classically considered to be short (<1 day) however, recent observations reported that the median peripheral blood (PB) human neutrophils lifespan is up to 10 times longer (5.4 days) (140–142). Neutrophils lifespan is further increased at sites of inflammation due to inhibition of cell apoptosis by inflammatory factors such as cytokines. Lifespan extension of neutrophils during inflammatory conditions may also alter neutrophils function and phenotype (143).

## Trauma and Neutrophil Phenotypes

Trauma and subsequent complications affect the phenotype and function of circulating neutrophils, and, particularly, in case of severe trauma, the development of dysfunctional neutrophils might play a detrimental role. Indeed, severe posttraumatic inflammation induces a boost in the release of banded and immature neutrophils into the circulation, leading to bone marrow exhaustion, and a compromised immune response, both associated with a poor outcome. Additionally, morphological changes were observed after trauma, including increased cell size and membrane plasticity and a modified shape, wherein neutrophils become more elongated (144). In trauma, there are immunosuppressive low-density neutrophils (LDNs), a subtype of neutrophil named after their discovery in the PB mononuclear cell (PBMC) (145, 146). These granulocytes are not only activated but express a high level of arginase activity, which in turn might be linked to T-cell function, providing a possible explanation for the impairment of the adaptive immunity mediated by neutrophils during trauma (146).

In sepsis, it has been demonstrated that this granulocyte subset inhibits T-cells, possibly via arginase release and/or ROS production (145, 147, 148). In contrast, there might be subsets of neutrophils, which are beneficial to repair the initial trauma impact. For example, a population of CD11b+/Gr-1+/CXCR4hi neutrophils likely recruited by vascular endothelial growth factor A (VEGF-A) induce revascularization via MMP-9 (149). While neutrophil heterogeneity is often described in the context of chronic inflammation, this is also seen in other scenarios such as cancer (145, 150).

## Neutrophil Phenotype and Function as a Biomarker for Patient Survival After Trauma

A quick and reliable prediction of prognosis is important particularly in the emergency room. Posttraumatic organ failure, is thought to be triggered by the initial inflammatory response (151). The time between the life span of peripheral neutrophils (5 days) and the time to produce new neutrophils from myelocytes (7 days) is critical in determining the risk of infectious complications such as septic shock (152). Early identification and prediction of septic shock may be greatly helpful in the identification of patients for the adaptation of the treatment regime (153, 154). Patients with subsequent MOF showed significantly higher mean circulating concentrations of C3a and thromboxane B2 at the first day post injury compared to the patients without MOF. Neopterin/creatinine ratios were also significantly higher in patients with multiple organ failure when MOF had already become established (155). So, seems to these mediators are useful for prediction of occurrence of secondary complications in traumatic patients but for realization more studies are needed.

Interestingly, in patients with major trauma, there was no significant difference in systemic C-reactive peptide and IL-6 levels between survivor and non-survivor groups. Furthermore, no differences between these groups were found for terminal complement complex, thromboxane B2, and neopterin/creatinine ratios (156). In contrast, some studies have shown plasma concentrations of neutrophil elastase, lactate, antithrombin III, IL-6, and IL-8 were significantly higher in non-survivors compared with survivors 24 h after trauma (151). Authors suggested that early alterations in serum levels of IL-6 constitute a useful predictive marker for identifying traumatic patients (156). Nevertheless, the role of IL-6 in critically ill patients has been discussed controversially in recent studies (157, 158). These data suggest that the overall level of the initial inflammatory response correlates with the development of post-traumatic organ failure.

Elevated serum NO and of its oxidation products (NOx) and of blood lactate in polytrauma patients are markers of a serious clinical course. However, a normal NOx combined with a very high lactate level may indicate a fatal prognosis in these patients (159). This suggests that elevated lactate levels may be an important prognostic factor rather than NOx itself. Decreased neutrophil responsiveness appears to be a prerequisite for septic shock after trauma. Indeed, the initial decreased responsiveness of circulating neutrophils to fMLP-induced FcγRII activation was related to the development of late onset septic complications after >5 days (14).

CD11b and active FcγRII/CD32 levels on unstimulated neutrophils, however, did not correlate with the severity of injury. Rather, there was a significant inverse correlation between the neutrophils ability to activate FcγRII in response to fMLP and the severity of injury. In addition, a decreased ability of fMLP to induce active FcγRII on neutrophils was found in patients who developed secondary complications of trauma such as SIRS or acute lung injury (ALI) and it seems to be a useful marker which could show occurrence of secondary complications (91).

For 9 of 10 septic shock patients, initial shock symptoms became evident between days 8 and 10 after admission. Measuring the kinetics of the neutrophil response demonstrated that the lowest neutrophil responsiveness to fMLP was found within the first 7 days after injury. Therefore, the impaired responsiveness to fMLP and other changes in receptor expression clearly preceded clinical symptoms of sepsis (160). On the other hand, CD64 expression level on day 1 is a fairly good predictor of outcome in critically ill patients with severe trauma and/or severe sepsis (132). Also, one study showed that CD64 expression level for neutrophils on postoperative day 1 is the best early predictor of intra-abdominal infection after colorectal cancer surgery (161).

Finally, the pre-operative surface expression of adhesion molecules involved in the migration of neutrophils such as CD99 and CD47 correlates with post-operative creatinine levels, a measurement of renal injury (162).

## NETs-Associated Predictive Markers in Trauma

Recent clinical studies have shown that the levels of circulating free-DNA (cf-DNA)/NETs can be potentially used for predicting injury severity following trauma with sepsis. It is important to note, however, that cf-DNA is not synonymous with NETs. The levels of cf-DNA/NETs in serum is increased in trauma patients who subsequently develop sepsis (133) whilst the levels of cf-DNA/NETs in synovial fluid are also increased in patients with septic arthritis (163). It is more recently used in the prediction of mortality in patients with severe burn injury (69).

DNase is naturally present in human blood and produced as a defense mechanism associated with NETs. The expression of DNase is increased in the early stages of sepsis after major trauma (69). DNase degrades NETs in a concentration dependent manner and DNase levels may be a potential biomarker of NETs formation (164).

Pentraxin 3 (PTX3) is a member of pentraxin family and acts as a soluble pattern recognition receptor (PRR) in the innate immune response (165). PTX3 is an extrahepatic acute-phase protein that has been implicated in the pathophysiology of trauma due to its extrahepatic formation and induction by different trauma-associated cytokines such as IL-1, IL-6 and TNF-α however this needs to be confirmed. Studies have shown that circulating PTX3 levels are associated with the injury severity and may reflect the immunological changes arising during soft tissue injury. Further studies are needed to prove PTX3 is a surrogate factor for soft tissue damage. PTX3 concentrations were higher in poly-traumatized compared to mono-traumatized and healthy individuals (166). PTX3 and some of the other components of NETs form a complex to enhance the actions of other NETs component proteins (164). Thus, new roles of PTX3 in the innate immune response, together with a pattern of binding to the NETs component proteins suggest an important role in NETosis (167).

## Conclusion

Neutrophils are main players in the context of inflammatory complications during and after traumatic injuries. Marked alterations in a range of neutrophils functions and in phenotypic markers occur following trauma, which causes a massive release of neutrophils, banded cells, and sometimes even immature cells from the bone marrow into the circulation. In trauma injury, neutrophils are able to modify the phenotype and function based on the body's requirements. In particular, CD11b is considered as important marker of poor prognosis (82) whilst the increased expression and activity of CXCR2 on neutrophils also correlates with neutrophil function and poor outcomes in ARDS (113). In this respect, the specific neutrophil phenotype and function could be considered as a biomarker of patient survival. Altered neutrophils phenotypes include increased expression of key cell surface molecules or enhanced life span and the expression of NETs and NETS-associated factors.

We conclude that neutrophils not only play a pivotal role in the regulation and modulation of trauma but that delineation of their particular phenotype, the expression of specific cell surface markers and the release of NETS-related factors could be used as a predictive tool for the management of trauma patients. Future studies should aim to identify key proteomic or transcriptomic markers that define each phenotype so that more rapid assessment of these can be made. Finally, randomized controlled studies using drugs directed against specific neutrophil subtypes will be essential to confirm this tenet.

## Author Contributions

EM, SZ, and MS wrote first draft. EM and IA revised the manuscript. SM, GF, JG, and IA has revised final version and added extra information.

### Conflict of Interest Statement

The authors declare that the research was conducted in the absence of any commercial or financial relationships that could be construed as a potential conflict of interest. The reviewer LL declared a shared affiliation, with no collaboration, with several of the authors, JG and GF, to the handling editor at time of review.

## Abbreviations

ALI: Acute Lung Injury
ARDS: Acute respiratory distress syndrome
CARS: Compensatory anti-inflammatory response
Cf-DNA: Circulating Free DNA
COX-2: Cyclooxygenase 2
DAMP: Damage-associated molecular pattern
ESL1: E-selectin ligand 1
FasL: Fas ligand
FI: phagocytosis index
fMLP: N-formyl-methionyl-leucyl-phenylalanine
FSAP: factor VII-activating protease
HMGB-1: High mobility group box-1
ISS: Injury Severity Score
LDN: Low-Density Neutrophils
Mac-1: alpha M beta 2 integrin
Mcl-1: Myeloid cell leukemia 1
MMP: Matric MetalloProteinase
MODS: Multiple organ dysfunction syndrome
MOF: Multiple organ failure
MPO: Myeloperoxidase
mtDNA: mitochondrial DNA
NADPH: Nicotinamide Adenine Dinucleotide Phosphate
NE: Neutrophil elastase
NETs: Neutrophil Extracellular Traps
NETosis: cell death characterized by release of decondensed chromatin and granular contents into the extracellular space
NF-κB: Nuclear Factor κB
NISS: New Injury Severity Score
NLRP3: NLR (NOD-like receptor) Family Pyrin Domain Containing 3
NO: Nitric Oxide
NOx: Oxidized NO products
NOS2: Inducible nitric oxide synthase or iNOS
PAF: Platelet-activating factor
PAMP: Pathogen-associated molecular pattern
PB: Peripheral Blood
PBMC: Peripheral Blood Mononuclear Cells
PECAM-1: Platelet endothelial cell adhesion molecule 1
PF4: Platelet Factor 4/CXC chemokine ligand 4
PG: Prostaglandin
PMA: phorbol-12-myristate-13-acetate
PMNE: Polymorphonuclear leukocyte elastase
PRR: Pattern Recognition Receptor
PSGL1: P-selectin glycoprotein ligand 1
PTX3: Pentraxin 3
ROS: Reactive oxygen species
SCI: Spinal cord injury
SIRS: Systemic inflammatory response syndrome
sL-selectin: soluble L-selectin
SOFA: Sequential Organ Failure Assessment
TBI: Traumatic brain injury
TGF-β: Transforming Growth Factor beta
TLR: Toll Like Receptor
TRALI: Transfusion-related acute lung injury
WHO: World Health Organization
VEGF-A: Vascular endothelial growth factor A.
